# Adverse Effects of Fluoroquinolones: A Retrospective Cohort Study in a South Indian Tertiary Healthcare Facility

**DOI:** 10.3390/antibiotics8030104

**Published:** 2019-07-27

**Authors:** Benitta Mathews, Ashley Ann Thalody, Sonal Sekhar Miraj, Vijayanarayana Kunhikatta, Mahadev Rao, Kavitha Saravu

**Affiliations:** 1 Department of Pharmacy Practice, Manipal College of Pharmaceutical Sciences, Manipal Academy of Higher Education, Manipal 576104, Karnataka, India; 2 Manipal Center for Infectious Diseases, Prasanna School of Public Health, Manipal Academy of Higher Education, Manipal 576104, Karnataka, India; 3 Department of Medicine, Kasturba Medical College and Hospital, Manipal Academy of Higher Education, Manipal 576104, Karnataka, India

**Keywords:** adverse effects, drug safety, FDA, fluoroquinolones

## Abstract

The Food and Drug Administration (FDA) safety review revealed that the use of fluoroquinolones (FQs) is linked with disabling and potentially permanent serious adverse effects. These adverse effects compromise the tendons, muscles, joints, nerves, and central nervous system of the human body. The purpose of the study was to investigate the incidence and risk factors for adverse drug reactions (ADRs) caused by FQs in comparison with other antibiotics used. A retrospective cohort study was conducted over seven months in Kasturba Medical College Hospital, Manipal, India. Patients who were prescribed with FQs were selected as the study cohort (SC; *n* = 482), and those without FQs were the reference cohort (RC; *n* = 318). The results showed that 8.5% (41) of patients developed ADRs in the SC, whereas 4.1% (13) of patients developed ADRs in the RC. With oral and parenteral routes of administration, almost a similar number of ADRs were observed. Levofloxacin caused the highest number of ADRs reported, especially with the 750-mg dose. Based on a multiple logistic regression model, FQ use (odds ratio (OR): 2.27; 95% confidence interval (CI): 1.18–4.39; *p* = 0.015) and concomitant steroid use (OR: 3.19; 95% CI: 1.31–7.79; *p* = 0.011) were identified as independent risk factors for the development of ADRs among antibiotics users, whereas age was found to be protective (OR: 0.98; 95% CI: 0.97–1.00; *p* = 0.047). The study found a higher incidence of ADRs related to FQs compared to other antibiotics. The study concludes a harmful association between FQ use and the development of ADRs. Moreover, FQs are not safe compared to other antibiotics. Hence, the use of FQs should be limited to the conditions where no other alternatives are available.

## 1. Introduction

For more than two decades, the safety of fluoroquinolones (FQs) was under investigation. These commonly used antibiotics are now advocated only when no alternatives are available, due to adverse effects, by recent warnings, for common conditions like uncomplicated urinary tract infections (UTIs) and acute bacterial infections of the sinus and bronchi [1]. Lomefloxacin, sparfloxacin, gatifloxacin, temafloxacin, grepafloxacin, and clenafloxacin were withdrawn from the market due to their fatal side effects [2,3]. Since 2008, the United States Food and Drug Administration (US FDA) mandated black-box warnings which were revised over the years. They were kept in place for warning the potential risks of tendinitis, worsening of myasthenia gravis, and peripheral neuropathy.

Even though FQs have great pharmacokinetic properties, there are concerns for human wellbeing. A few unfavourable responses were accounted by FQs in the final phases of clinical trials, and most of these included the skin, musculoskeletal, hepatic, gastrointestinal tract (GIT), and central nervous systems (CNS) [4]. Modifications in the FQ structure may be responsible for the development of adverse drug reactions (ADRs) such as phototoxicity, prolonged corrected Q-wave to T-wave (QT) interval (QTc), and CNS adverse effects [5,6]. FQs are lipoidal in nature, which accounts for their increased affinity in both cartilage and bone. This property can ultimately affect the tendons, muscles, and joints. The possible underlying mechanism for muscle pain and muscle weakness may be cellular apoptosis. The most severe ADRs associated with FQs are tendinitis or tendon rupture, while other reactions include joint pain, myalgia, arthralgia, neck pain, and muscle spasms.

The varying affinity for the γ-aminobutyric acid (GABA) receptor determines the likelihood of seizure occurrence. CNS stimulation, which results from GABA displacement by the FQs from their receptor, can trigger various symptoms, including anxiety, hallucination, paranoia, delirium, tremors, and insomnia [7]. Warnings were given on the possible dizziness which can occur from ciprofloxacin (CFX) and moxifloxacin (MFX). The development of dizziness was also reported after the levofloxacin (LFX) use [8,9,10]. Other symptoms that can be precipitated due to increased cerebrospinal fluid (CSF) pressure around the brain include headache, lightheadedness, and double vision. Dermatological problems are another frequently reported ADR with FQ use. Symptoms like rash and pruritus can develop even after administration of a single dose of the drug.

In 2004, warning labels of FQs included peripheral neuropathy, which is characterized by numbness, tingling, or pricking sensation [1]. It is perceived to be caused mainly due to systemic FQs. The symptoms of peripheral neuropathy are usually quick and appear within a couple of days after use, but its identification may be delayed due to its vague clinical manifestation [11]. In some instances, the adverse effects progressed for over a year, regardless of stopping the medication [12]. A nested case-control study reported that the use of oral FQs was associated with a higher risk of peripheral neuropathy, which depends on the exposure time and cumulative dose [13]. A survey on FQ-associated adverse event cases posted online and a case series contributed significantly to the FDA label changes. The survey established a conceivable relationship between FQ use and adverse effects involving the peripheral nervous system (PNS). The latter highlighted that healthy individuals developed delayed reactions after FQ use. These reactions led to severe impairment of multiple organ systems [14,15]. Few case reports suggest that FQs such as CFX, LFX, and MFX cause syndrome of inappropriate antidiuretic hormone (SIADH) [16,17,18]. This mechanism is likely to be due to the involvement of GABA and *N*-methyl-d-aspartate (NMDA) receptors, which leads to the increased release of anti-diuretic hormone (ADH), which in turn causes water retention, thereby prompting hyponatremia [19].

The benefits of FQs showed that these were proved to be useful in both ocular infections and surgical prophylaxis in ophthalmology. However, there were reports of FQs causing eye injury, varying from eye pain to damage to the ocular muscles and retina. Additionally, there were reports on visual impairments. Double vision can occur due to an impairment of coordination between nerves of both eyes and an increase in intraocular pressure [20].

Nausea and vomiting are the most common gastrointestinal adverse reactions. Similar to other antibiotics, FQs can cause *Clostridium difficile*-associated diarrhea (CDAD) due to alteration of the gut’s flora, which ultimately promotes the growth of *Clostridium difficile*. Inhibition of potassium channels in the mechanism is mainly responsible for QTc prolongation and arrhythmia. Sparfloxacin and grepafloxacin were removed from the market due to their cardiotoxic properties [5]. Also, other symptoms that were reported include hepatotoxicity, alteration of blood glucose levels, and renal injury. Recently, the FDA warned against the use of systemic FQs. However, the majority of the studies conducted on FQ safety were from the Western population. Extrapolating Western data in South Asians, especially in Indians, may not be pragmatic. There are differences among regions or even countries in the occurrences of ADRs due to differences in diseases, prescribing practices, genetics, diet and tradition of the people, drug distribution, use indications, dose, availability, and regulatory policies. Similarly, there is a paucity of evidence regarding the disabling adverse effects of FQs in the Asian populace. Almost all antibiotics, including FQs, are easily accessible to the public in India. However, stronger warnings were not implemented regarding their usage. Therefore, this study was designed to investigate the risk of the development of adverse effects following FQ use and to determine whether their route of administration, dose, and duration play a significant role in the development of adverse effects.

## 2. Results

### 2.1. Characteristics of the Study Population

A total of 800 patients admitted to hospital from January to April 2016 were enrolled in the present study. The study cohort (SC) comprised 482 patients with FQ prescriptions, and the reference cohort (RC) consisted of 318 patients with antibiotics other than FQs. A total of 8.5% (41) of the 482 in the SC developed ADRs, whereas, in the RC, only 4.1% (13) of 318 patients developed ADRs. In the patients who developed ADRs, a higher incidence was observed among males in both the SC (78.1%) and RC (53.9%). The incidence of ADRs was predominantly higher in the age group of 61–70 years for both the SC (24.4%) and RC (30.8%) (Table 1).

### 2.2. Prescription Details of the Antibiotics

#### 2.2.1. Prescription Details of Fluoroquinolones

**Figure 1:**
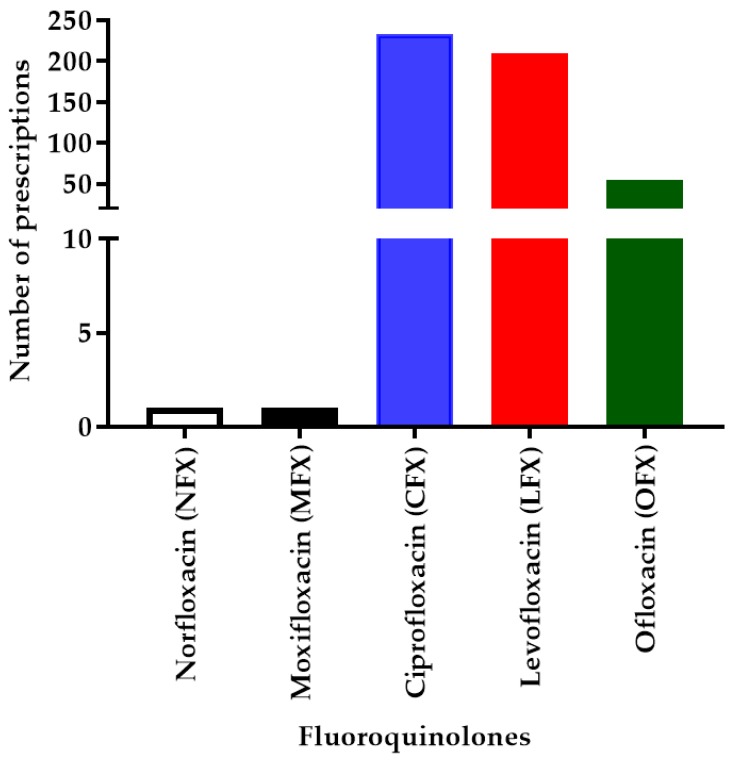
Number of fluoroquinolone (FQ) prescriptions (*N* = 498). This chart represents different FQs prescribed in the study population with the number of prescriptions for each FQ, indicating that ciprofloxacin (CFX) has the highest number of prescriptions followed by levofloxacin (LFX), ofloxacin (OFX), moxifloxacin (MFX), and norfloxacin (NFX).

In our study setting, mainly five types of FQs were prescribed, which include CFX, LFX, ofloxacin (OFX), MFX, and norfloxacin (NFX). A total number of 482 patients in the SC received 498 FQ prescriptions. Out of the 498 FQs prescribed, irrespective of the different doses and routes, CFX (46.79%; 233) was found to be the most common, followed by LFX (41.9%; 209), OFX (10.8%; 54), MFX (0.2%; 1), and NFX (0.2%; 1) (Figure 1).

**Figure 2:**
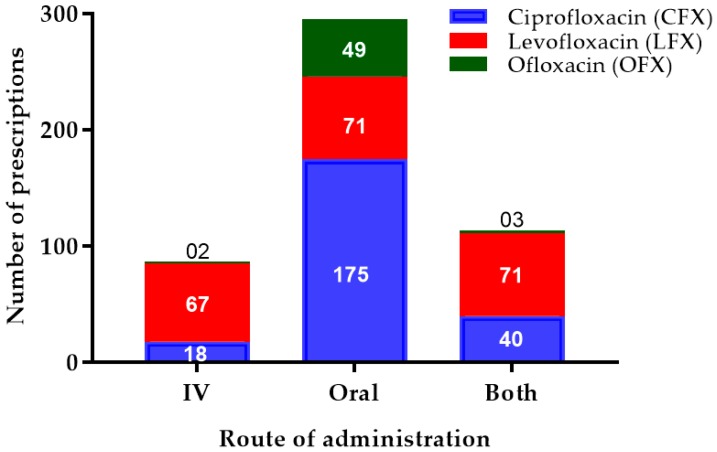
Number of different fluoroquinolone prescriptions with their route of administration. This chart represents the three major types of FQs (CFX, LFX, OFX) prescribed in the study population with their route of administration, indicating that the oral route was the most commonly prescribed for all the FQs. IV—intravenous.

Apart from the intravenous (IV) or oral route only, some patients received both routes of FQs. The oral route was the most commonly preferred route for CFX (75.1%; 175), LFX (33.9%; 71), and OFX (90.7%; 49). LFX administrations were almost similar in all routes of administration, i.e., IV (32.1%; 67), oral (33.9%; 71), and both (33.9%; 71) (Figure 2).

#### 2.2.2. Prescription Details of Antibiotics Other Than Fluoroquinolones

A total number of 318 patients in the RC received 564 other antibiotic prescriptions, which included 25 different antibiotics. Few patients were administered with more than one antibiotic. The number of other antibiotic prescriptions is given in Table 2.

### 2.3. Adverse Drug Reactions of Antibiotics

**Figure 3:**
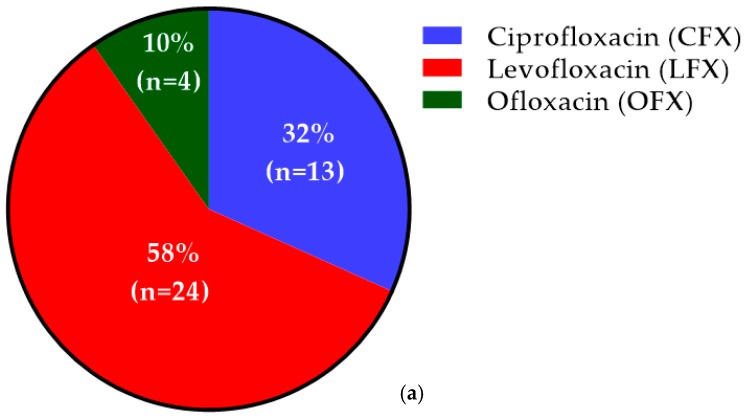

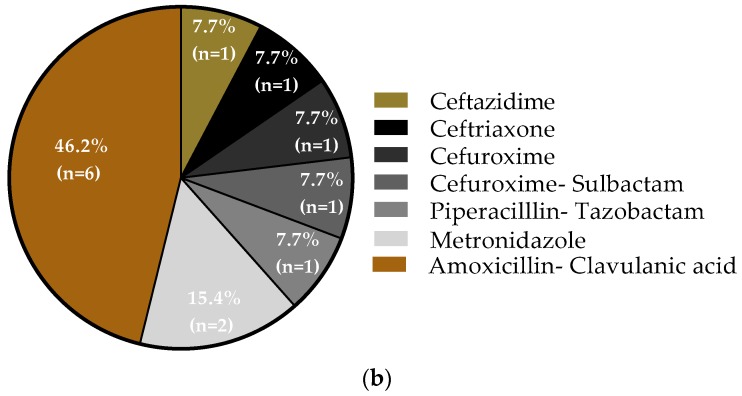
Incidence of patients who developed adverse drug reactions (ADRs) among both cohorts: (**a**) incidence of patients who developed ADRs among the study cohort, indicating the highest incidence of 172 (58%) with LFX; (**b**) incidence of patients who developed ADRs among the reference cohort, indicating the highest incidence of 173 (46.2%) with amoxicillin–clavulanic acid.

Out of 41 patients who developed ADRs with FQs, LFX caused ADRs in 58% (24) of patients, whereas CFX and OFX caused ADRs in 32% (13) and 10% (4) of patients, respectively (Figure 3a). Based on the number of prescriptions of each FQ, the incidence rate of ADRs among LFX, OFX, and CFX was found to be 11.5%, 7.4%, and 5.6%, respectively.

Out of 13 patients who developed ADRs in RC, ceftazidime, cefuroxime, ceftriaxone, cefuroxime–sulbactam, and piperacillin–tazobactam caused ADRs in only 7.7% (1) of patients each. On the other hand, amoxicillin–clavulanic acid and metronidazole caused ADRs in 46.2% (6) and 15.4% (2) patients, respectively (Figure 3b).

#### 2.3.1. ADRs Based on the Route of Administration of Fluoroquinolones

**Figure 4:**
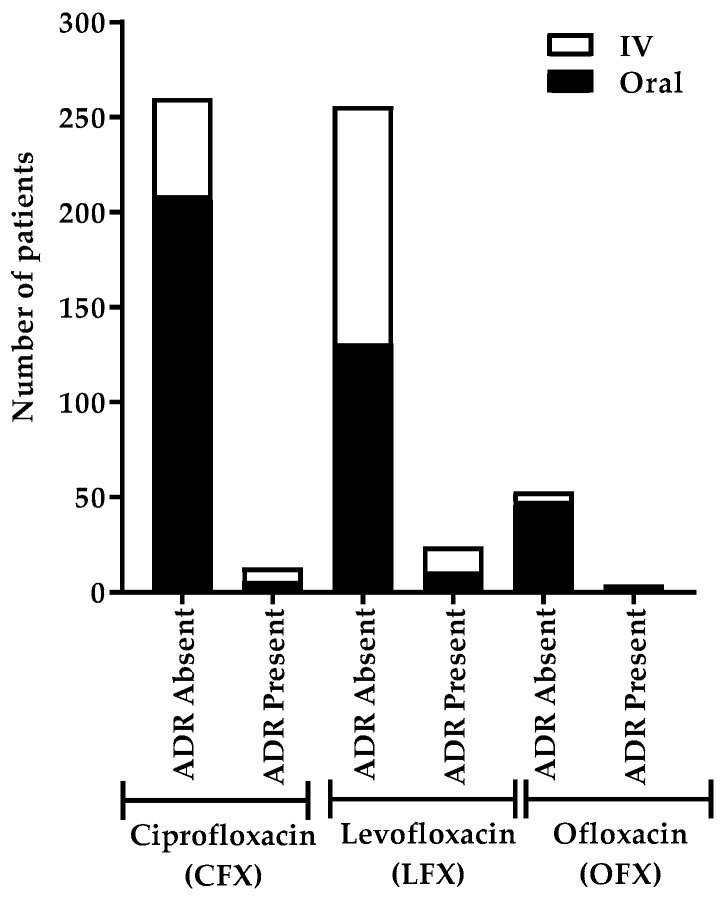
Number of patients who developed ADRs based on the route of administration of different fluoroquinolones. This chart shows the incidence of patients who developed ADRs after the administration of different types of FQs via different routes (IV and oral), indicating the highest incidence with IV LFX administrations.

The highest number of patients developed ADRs after IV LFX administration (Figure 4). Nearly an equal number of patients developed ADRs with both IV and oral administration of LFX and CFX. No ADRs were developed in patients after IV OFX administration.

#### 2.3.2. ADRs Based on the Dose of Fluoroquinolones

Only 2.9% (6) of patients developed ADRs with oral CFX administration, which was reported for the use of a 500-mg dose. This dose, 500 mg, was the most frequently used LFX dose for the oral and IV routes, which accounted for ADRs in 5.9% and 8.9% patients, respectively. Most importantly, 75% (3) of patients who received oral LFX developed ADRs. No ADR was developed after an IV dose of OFX, whereas 7.8% (4) of patients developed ADRs after oral OFX administration (Table 3).

#### 2.3.3. ADRs Based on the Duration of the Use of Antibiotics

**Figure 5:**
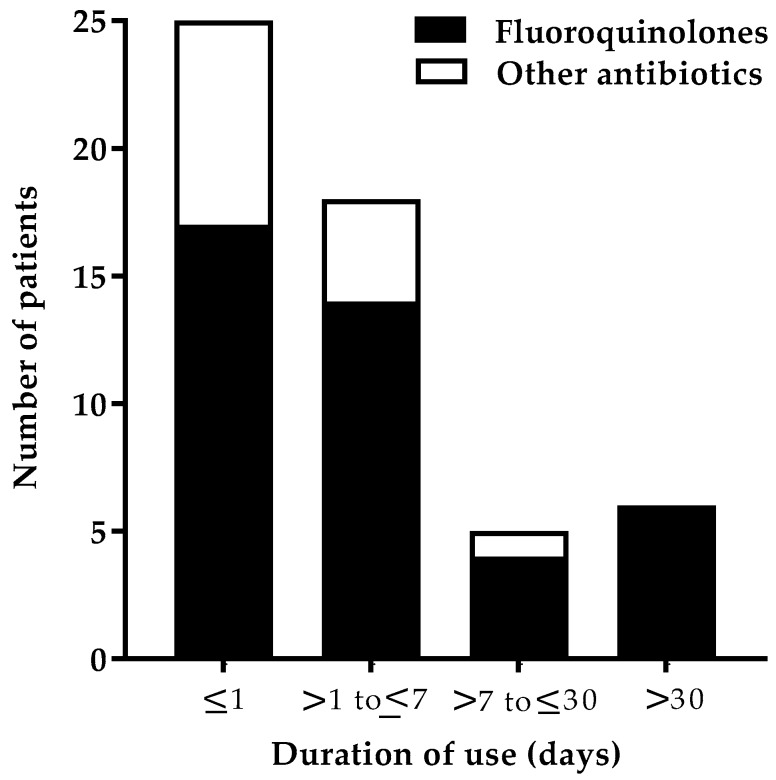
Number of patients who developed ADRs based on the duration of use of antibiotics. This chart shows the incidence of patients who developed ADRs based on the duration of use of both FQs and other antibiotics. Duration of the use of the antibiotic was categorized as “less than or equal to one day” (≤1), “up to one week” (>1 to ≤7), “up to one month” (>7 to ≤30), and “more than one month” (>30).

The onset of the ADR symptoms following the initiation of treatment was mostly seen within a day (Figure 5). The onset of the ADRs can be classified into immediate (minutes–one hour after exposure) and delayed (24–48 h after exposure). After FQ use, 29.3% (12) of patients developed an immediate reaction, whereas delayed reactions occurred in 70.7% (29) of patients.

#### 2.3.4. Classification of Antibiotic-Related ADRs

In the SC, a total of 59 ADRs were experienced by 41 patients; 13 patients were seen with more than one ADR (Table 4). Dermatological (20.3%) symptoms, along with others (20.3%), were mainly seen. Itching, rashes, and erythema fall under the dermatological system, whereas other body systems involve breathlessness, hyperglycemia, hyponatremia, SIADH, and vision problems. In the RC, a total of 17 ADRs were experienced by 13 patients.

### 2.4. Risk Determination

Univariate analysis was used to identify the risk factors associated with the incidence of ADRs among two cohorts. Multiple logistic regression was used to develop the ADR prediction model and to calculate the odds ratio (OR) among antibiotics users. The dependent variable used in the analysis was the presence of ADRs, and independent variables included in the analysis were FQ use, age, and concomitant steroid use (Table 5). The FQ use (OR: 2.27; confidence interval (CI): 1.18–4.39; *p* = 0.015) and concomitant steroid use (OR: 3.19; 95% CI: 1.31–7.79; *p* = 0.011) were identified as independent risk factors for the development of ADRs among antibiotics users by multiple logistic regression, whereas age was found to decrease (protective) the incidence of ADRs (OR: 0.98; 95% CI: 0.97–1.00; *p* = 0.047).

Using multiple logistic regression coefficients, an ADR prediction model was developed.

The logistic regression model obtained for our data was as follows:

$$p(ADR)=\frac{e^{-2.38+Age\times -0.017+FQ\times 0.82+Steroid\times 1.16}}{1+e^{-2.38+Age\times -0.017+FQ\times 0.82+Steroid\times 1.16}}.$$

**Figure 6:**
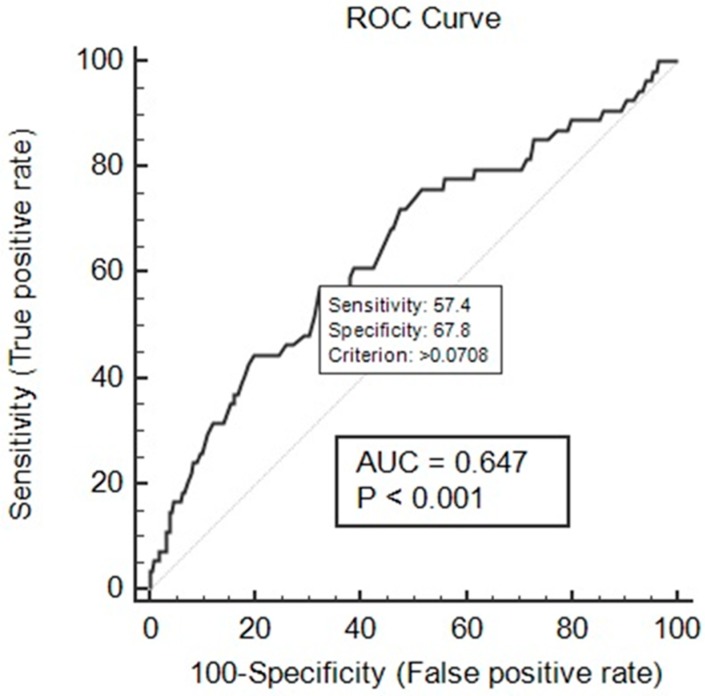
Receiver operating characteristic (ROC) curve of the developed ADR prediction model. The ROC curve analysis of the developed prediction model exhibited good discriminating power for the developed prediction model with area under the curve (AUC) = 0.647 at *p* < 0.001.

The performance of the developed ADR prediction model was evaluated by the Hosmer and Lemeshow test, receiver operating characteristic (ROC) curve analysis, and residual analysis. The Hosmer and Lemeshow test showed a good model fit at chi-square = 8.95 and *p*-value = 0.346 (indicating a statistically insignificant difference between predicted and observed probability). The ROC curve analysis showed good discriminating power for the developed prediction model with area under the curve (AUC) = 0.647 at *p* < 0.001 (Figure 6).

**Figure 7:**
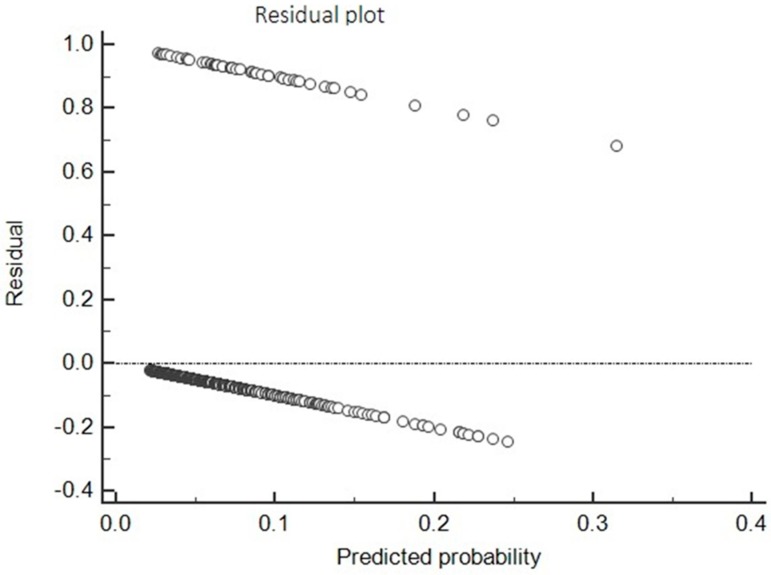
Residual plot of the developed ADR prediction model. The model exhibited a relatively equal distribution of points above and below at the horizontal line at residuals = 0, indicating non-violation of the assumption of linearity and equal variance of the regression model.

The residual plot (Figure 7) of the developed model showed a relatively equal distribution of points above and below at the horizontal line at residuals = 0, indicating non-violation of the assumption of linearity and equal variance of the regression model.

### 2.5. Causality Assessment of the ADRs Related to the Antibiotics

In the SC, most of the ADR cases (60.9%; 25) were categorized as possible causality based on the World Health Organization (WHO) Uppsala Monitoring Center (UMC) criteria scale (Table 6). While using the Naranjo probability scale, a maximum number of ADR cases (60.9%; 25) were classified under the probable category. The majority of the ADR cases (65.9%; 27), according to Hartwig’s severity assessment scale, were found to be of moderate severity (level 3, level 4a, and 4b).

In the RC, not much difference was observed between the probable (46.2%) and possible (53.9%) categories based on the WHO–UMC criteria scale (Table 7). While using the Naranjo probability scale, an equal number of ADR cases (46.2%) were classified under the probable and possible categories. The majority of the ADR cases, according to Hartwig’s severity assessment scale, were found to be of moderate (69.2%) severity.

## 3. Discussion

The present study addresses the rarity of data on the safety attributes of FQs in the Indian setting. This is very important as resistance to FQs among various pathogens increased in India, while quinolone consumption is also high and rising in India [21]. In this study, the incidence of ADRs among FQ users was higher compared to other antibiotics users (8.5 vs. 4.1) and more in the age group of 61–70 years for both groups. A Japan study reported an increased incidence of ADRs among children (<15 years) and elderly people (≥75 years) [22]. This may be because the mean age of our study population was 50.9 ± 17.8 years. We observed a male predominance in the occurrence of the ADRs among FQs users (78.1%) and other antibiotics users (53.9%), since a majority of the subjects were male (67.4% vs. 63.8%) in both groups. This may be because gender differences exist concerning health-seeking behavior in developing countries, indicating that women utilize formal healthcare to a lesser extent compared to men [23]. On the other hand, in a study by Lapi et al., females (53.8%) accounted for a more significant portion of the ADRs [24].

We found that a majority of the ADRs were due to LFX followed by CFX and OFX. This may be due to common ADRs such as nausea, vomiting, and diarrhea, which are mainly associated with LFX. Previous studies by Oreagba et al. and Jose et al. also showed a similar predominance of LFX [4,25]. Many FQs are available in IV, as well as formulations, providing flexibility in administration and offering the potential for IV–oral switch, once the patient improves. However, in our findings, the highest number of patients developed ADRs after IV LFX administration. Although the maximum number of ADRs was developed with 500 mg of oral LFX, the highest incidence (75%) of ADR was among high-dose (750 mg) oral LFX users. Piscitelli et al. reported an overall incidence of any ADR as 95% for oral 750-mg LFX-treated patients [26]. However, there is a paucity of studies that associate the dose of FQs and ADRs [27].

The majority of the ADRs, as a function of the duration of FQ therapy, were found to be ≤1 day (41.5%, 17) and within a week (34.1%, 14). On the contrary, in a study of the Nigerian population, only 13.6% experienced the ADR in <1 day, and the majority of the onset of ADRs (74.5%) was seen within 1–10 days of the start of FQ therapy [4]. The most common organ systems affected were dermatological and others (each 20.3%) after FQ use. Similarly, in another study, dermatological reactions (25%) were the most reported adverse reactions [28]. In a study conducted by Jose et al., a higher proportion of reactions was reported to involve the dermatological system than in our study [20]. CFX was found to be responsible for most skin reactions, as seen in other studies [28,29]. Only 6.8% of the patients experienced musculoskeletal adverse effects in our study. There is robust evidence regarding the association between LFX and tendon injury [15]. Several case reports showed the development of tendinitis following LFX treatment [30,31,32]. The FDA reported that musculoskeletal adverse effects occur in 97% of the cases. This variation in result may be due to differences in the inclusion criteria of the study population.

The percentage of adverse reactions involving the CNS (11.9%) in our study was found to be comparable to the results obtained by Leone et al. (12.2%) [28]. In this study, CFX (1.7%) was associated with an effect on the PNS. According to a study by Etminan et al., FQs showed a higher risk of causing peripheral neuropathy (relative risk (RR) = 1.83, 95% CI 1.49–2.27) [33]. A case report published by Francis et al. presented a case with CFX-induced peripheral neuropathy [11]. The increased reports of neurological-related ADRs to FQs compared to other antibiotics may be due to a periodic warning by the FDA and recent changes in the labeling of the FQ class. Ptosis was seen in one of the patients who was administered with OFX. Jones et al. found an association of FQs with ptosis [19]. A total of 4.7% (10) of patients who were administered with LFX experienced adverse effect related to the cardiovascular system (CVS), of which five symptoms were related to arrhythmia. A systematic review and meta-analysis by Liu et al. reported that MFX and LFX had a higher chance of causing irregular heartbeat [34]. In a randomized trial involving elderly patients, MFX (8.3%) and LVX (5.1%) were shown to develop adverse cardiac problems [35]. A review article by Rubinstein et al. suggested that FQs are safe, although they require surveillance in the presence of any cardiac disease, cardiac drug, or electrolyte imbalance [5]. A population-based study by Lapi et al. suggested that the use of LFX was safe and MFX, CFX, and gatifloxacin were seen with severe dysrhythmia [36]. In our study, only one case of nausea (2.4%) was reported, and CFX was responsible for this reaction. According to a study by Chodosh et al., nausea was observed in 27 out of 213 (12.8%) people using CFX [37].

In our study, hyperglycemia was observed following the use of OFX in a diabetic patient. According to a study by Chou et al., FQs had a higher risk of causing both hyperglycemia and hypoglycemia in diabetic patients [38]. Warnings of altered blood glucose level were issued on OFX use in patients with an antidiabetic agent [39]. Of the 28 respiratory tract infection patients treated with FQs, 23 received LFX, and only four received OFX. ADRs were seen only in three patients taking LFX (13%). In a double-blind study, the extent of side effects was slightly higher in patients taking OFX (11.1%) than those taking LFX (6.4%) [34]. In our study, CFX caused hyponatremia and SIADH in a 58-year-old man, which was similar to the case reported by Mancano et al. [16]. The author suggested that the elderly were at higher risk (66–73 years). In other case reports, patients developed hyponatremia and SIADH due to MFX and LFX [17,18]. The ADRs developed in 17.1% (7) of patients who were concurrently administered with systemic steroids. A report by Khaliq and Zhanel showed that 40 patients who received FQs concurrently with steroids developed tendon rupture [40]. In another study, the occurrence of tendinitis with concomitant steroid use was reported as 31% [28].

Using the WHO–UMC criteria scale, the highest number of FQ-related adverse reactions were in the possible (60.9%) category. According to the Naranjo probability scale, the reactions were mainly categorized as probable (60.9%), whereas contrasting results were seen in other studies [25,28]. The ADRs reported on FQs use were of moderate (65.9%; 27) severity. According to Oreagba et al., ADRs of mild severity were relatively more frequent than ADRs of moderate severity [4]. In our study, the incidence of ADRs was observed more among FQs (8.5%) compared to other antibiotics (4.1%). FQ use had a higher risk of causing ADRs as compared to other antibiotics (OR = 2.391, 95% CI: 1.245, 4.592). There is a harmful association between FQ use and development of ADRs (*p* = 0.015). Contrasting results were noted from various studies in different tertiary care hospitals across India. Shamna et al. and Jayanthi et al. reported a higher incidence of ADRs using cephalosporin than FQs [41,42]. Dhar et al. reported a higher incidence of ADRs with beta-lactams and aminoglycosides [43].

Univariate and multivariate analyses were used to find the association between risk factors and the incidence of ADRs, and adjusted OR was calculated. Based on multiple logistic regression, an ADR prediction model was developed. FQ use and the concomitant use of steroid were independent risk factors, and age was a protective factor for the development of ADRs. The developed model was evaluated using the Hosmer and Lemeshow test, ROC curve, and residual analysis. The developed model showed an acceptable match between predicted ADRs and observed ADRs (chi-square = 8.95; *p*-value = 0.346). The non-significant *p*-value for the Hosmer and Lemeshow measure demonstrated that the observed rates were statistically the same across the risk group defined by the test, which shows that the model had a good fit. The ROC curve analysis was performed for predicted probability and occurrences of ADRs. It showed good discriminating power for the developed prediction model of 0.647 at *p* < 0.001. The residual analysis showed that the model can predict the presence and absence of ADR outcome.

This study highlights the importance of spontaneous reports of adverse effects of FQs. The research made an interesting comparison with other classes of antibiotics in terms of cohort event reporting of ADRs. The observations should encourage healthcare professionals and stakeholders at the institutional and the national levels to conduct periodic antibiotic utilization audits. The findings reveal the necessity of antibiotic stewardship and educational intervention. These are important to optimize FQ prescriptions and for sustainable behavior changes of clinicians toward rational antibiotic use, especially in developing countries. Since this was a retrospective cohort study, the investigators did not have access to other parameters which might influence the development of ADRs. This is one of the major limitations of the study. Apart from this, certain ADRs, especially those of mild severity, may be overlooked due to the retrospective nature of the study design as compared to a prospective study. Moreover, missing information related to adverse events interfere with the causality assessment of ADRs.

## 4. Materials and Methods

**Figure 8:**
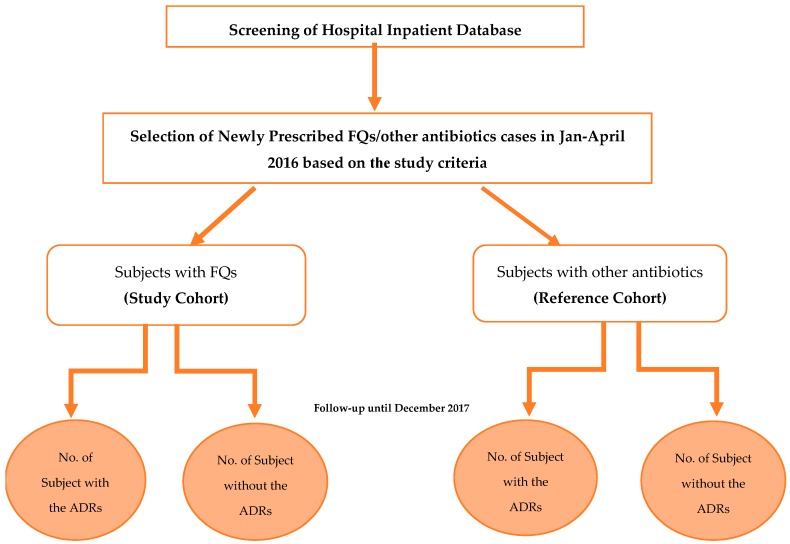
Study flow chart.

A retrospective cohort study was carried out for seven months (September 2017 to March 2018) in Kasturba Hospital, Manipal, a 2032-bed tertiary care teaching hospital in Udupi District, Karnataka, India. The study was carried out after receiving ethical approval from the Institutional Ethics Committee (IEC) of Kasturba Hospital, Manipal (IEC reference No. 578/2017 dated 13 September 2017). The data collections were carried out following the rules of the Declaration of Helsinki of 1975, revised in 2013. A total of 800 patients admitted in various departments of the hospital from January 2016 to April 2016 were included. These patients were categorized into two cohorts: study cohort (SC) and reference cohort (RC) (Figure 8). The SC included patients (*n* = 482) who were prescribed with FQs, and the RC included patients (*n* = 318) who were prescribed with antibiotics other than FQs. In-patients of both genders, irrespective of the age, who were newly prescribed with FQs or other antibiotics for treatment or prophylactic use for infectious diseases, were included. Out-patient cases, those with incomplete medical records, and patients prescribed a combination of FQs or together with other antibiotics were excluded.

Demographical details were recorded along with the clinical symptoms and lab investigations. Details regarding the diagnosis, route of administration, dose, frequency and duration of antibiotics, supportive treatment provided, duration of hospitalization, and ADRs such as symptoms and onset details were collected. Causality assessment for the ADRs was performed by the Naranjo probability scale (definite, probable, possible, or unlikely) and the World Health Organization Collaborating Center for International Drug Monitoring, the Uppsala Monitoring Centre (WHO–UMC) criteria scale (certain, probable/likely, possible, unlikely, unclassified, or unclassifiable). Hartwig’s severity assessment scale was used to classify the severity of reaction into mild (levels 1 and 2), moderate (levels 3, 4a, and 4b), or severe (levels 5, 6, and 7).

### 4.1. Sample Size

By using the comparison of the proportion method, the sample size was estimated with 80% power and 5% level of significance as 800 consisting of both cohorts.

### 4.2. Statistical Analysis

Data analysis was performed using the Statistical Package for Social Sciences (IBM SPSS) version 20.0. Cross-tabulation was used to calculate the incidence of ADRs across the different routes of administration, dose, and duration of FQs. Initially, univariate analysis was used to identify the risk factors associated with the incidence of ADRs among the groups. Multiple logistic regression was performed to analyze the association between risk factors and the incidence of ADRs, and to obtain an adjusted odds ratio at 95% CI. A *p*-value <0.05 was considered statistically significant for all the statistical analysis. The developed multiple logistic regression model was evaluated using the Hosmer and Lemeshow test, ROC curve, and residual analysis. GraphPad Prism version 7.0 was used to generate the graphs.

## 5. Conclusions

FQ use has an increased risk of causing adverse reactions. This study concluded a harmful association between FQ use and the development of ADRs. This study is the first of its kind, reporting the incidence of FQ-related ADRs based on cohort event reporting, and developing a prediction model for the development of ADRs after FQ use. Our findings showed that the incidence of ADRs was more following FQ use than the use of other antibiotics for the same conditions. The physician should take into consideration the safety profile of FQs before prescribing them to the patients. The use of FQs should be restricted to cases in which alternatives are not available.

## Figures and Tables

**Table 1:** Characteristics of the study population (*n* = 800).

Patient Characteristics	Study Cohort (*n* = 482)		Reference Cohort (*n* = 318)	
	ADR Present	ADR Absent	ADR Present	ADR Absent
Number of patients	8.5% (41)	91.5% (441)	4.1% (13)	95.9% (305)
Age (years), mean ± SD	50.9 ± 17.8	56.4 ± 15.9	49.5 ± 17.5	49.3 ± 16.6
Gender				
Male	78.1% (32)	66.4% (293)	53.9% (7)	64.3% (196)
Female	21.9% (9)	33.6% (148)	46.1% (6)	35.7% (109)
Age group				
0–10	-	0.2% (1)	-	0.7% (2)
11–20	4.9% (2)	1.4% (6)	7.7% (1)	2.3% (7)
21–30	12.2% (5)	6.1% (27)	7.7% (1)	12.8% (39)
31–40	14.6% (6)	11.6% (51)	15.4% (2)	16.1% (49)
41–50	14.6% (6)	12.9% (57)	23.1% (3)	21.3% (65)
51–60	19.5% (8)	22.2% (98)	7.7% (1)	18.7% (57)
61–70	24.4% (10)	26.1% (115)	30.8% (4)	18.4% (56)
71–80	7.3% (3)	15.6% (69)	7.7% (1)	7.9% (24)
81–90	2.4% (1)	3.6% (16)	-	2.0% (6)
91–100	-	0.2% (1)	-	-
Duration of hospitalization (days), median (IQR)	9.0 (15–5)	5.0 (10–4)	8.0 (13–6.5)	6.0 (9–4)

ADR: adverse drug reaction; IQR: interquartile range.

**Table 2:** Number of prescriptions of antibiotics other than fluoroquinolones in the reference cohort.

Serial No	Other Antibiotics	Number of Prescriptions (% (No.))
1	Amoxicillin	0.5 (3)
2	Amoxicillin–clavulanic acid	18.4 (104)
3	Amikacin	0.7 (4)
4	Ampicillin–cloxacillin	0.2 (1)
5	Azithromycin	4.9 (28)
6	Cefixime	2.3 (13)
7	Cefazolin	0.2 (1)
8	Cefpodoxime	1.6 (9)
9	Cefoperazone	1.9 (11)
10	Cefoperazone–sulbactam	13.8 (78)
11	Ceftazidime	0.4 (2)
12	Cefuroxime	7.3(41)
13	Cefuroxime–clavulanic acid	2 (0.4)
14	Cefuroxime–sulbactam	9.9 (56)
15	Ceftriaxone	10.7(60)
16	Ceftriaxone–sulbactam	0.4 (2)
17	Clindamycin	1.2 (7)
18	Doxycycline	1.4 (8)
19	Gentamycin	1.4 (8)
20	Linezolid	0.5(3)
21	Meropenam	1.6 (9)
22	Metronidazole	5.3 (30)
23	Piperacillin–tazobactam	5.1 (29)
24	Sulfamethoxazole–trimethoprim	1.4 (8)
25	Tinidazole	8.3(47)
Total		100 (564)

**Table 3:** Number of patients who developed ADRs based on the dose of fluoroquinolones.

	Route	Dose (mg)	ADR Present	ADR Absent	Total
**Levofloxacin**	Oral	250	0	100% (2)	0.3% (2)
		500	5.9% (8)	94.1% (128)	22.3% (136)
		750	75% (3)	25% (1)	0.7% (4)
	IV	250	0	100% (1)	0.2% (1)
		500	8.9% (11)	91.1% (113)	20.3% (124)
		750	15.4% (2)	84.6% (11)	2.1% (13)
**Ciprofloxacin**	Oral	200	0	100% (1)	0.2% (1)
		400	0	100% (1)	0.2% (1)
		500	2.9% (6)	97.1% (203)	34.3% (209)
		750	0	100% (2)	0.3% (2)
		1000	0	100% (2)	0.3% (2)
	IV	200	11.9% (5)	88.1% (37)	6.9% (42)
		250	0	100% (1)	0.2% (1)
		400	0	100% (1)	0.2% (1)
		500	0	100% (4)	0.7% (4)
		1000	20% (2)	80% (8)	1.6% (10)
**Ofloxacin**	Oral	200	6.3% (1)	93.7% (15)	2.6% (16)
		400	8.6% (3)	91.4% (32)	5.7% (35)
		450	0	100% (1)	0.2% (1)
	IV	200	0	100% (3)	0.5% (3)
		400	0	100% (2)	0.3% (2)

IV: intravenous.

**Table 4:** ADR classification based on the related organ system.

Organ Systems	ADRs among FQ Users (*N* = 59)	ADRs among Other Antibiotics Users (*N* = 17)
Gastrointestinal	15.3% (9)	41.2% (7)
Dermatological	20.3% (12)	17.6% (3)
Musculoskeletal	6.8% (4)	0
CNS	11.9% (7)	23.5% (4)
PNS	6.8% (4)	0
Cardiovascular	18.6% (11)	5.9% (1)
Others	20.3% (12)	11.8% (2)

ADRs: adverse drug reactions; CNS: central nervous system; FQs: fluoroquinolones; PNS: peripheral nervous system.

**Table 5:** Univariate and multivariate analysis of factors associated with the incidence of ADRs in FQ users and antibiotics other than FQ users.

Factor	Univariate Analysis			Multivariate Analysis		
	Unadjusted OR	95% CI	*p*-Value	Adjusted OR	95% CI	*p*-Value
Age	0.99	0.97–1.01	0.212	0.98	0.97–1.00	0.047 *
Male	1.37	0.74–2.53	0.319	---	---	---
Fluoroquinolones	2.18	1.15–4.14	0.017 *	2.27	1.18–4.39	0.015 *
Concomitant steroid use	3.32	1.39–7.93	0.007 *	3.19	1.31–7.79	0.011 *
Duration of antibiotics use (days)	1.02	0.96–1.09	0.528	---	---	---

CI: confidence interval; OR: odds ratio; *p*: probability; * statistically significant.

**Table 6:** Causality assessment of ADRs in the study cohort. WHO–UMC—World Health Organization Uppsala Monitoring Center.

Scale	Causality Assessment	Fluoroquinolones			Total
		Ciprofloxacin	Levofloxacin	Ofloxacin	
**WHO–UMC Criteria**	Certain	0	1	0	2.4% (1)
	Probable/Likely	6	8	1	36.6% (15)
	Possible	7	15	3	60.9% (25)
**Naranjo Probability**	Definite	0	1	0	2.4% (1)
	Probable	11	11	3	60.9% (25)
	Possible	2	12	1	36.6% (15)
**Hartwig’s Severity Assessment**	Level 1	1	2	0	7.3% (3)
	Level 2	1	6	1	19.5% (8)
	Level 3	5	13	2	48.8% (20)
	Level 4a	1	0	0	2.4% (1)
	Level 4b	3	2	1	14.6% (6)
	Level 5	1	0	0	2.4% (1)
	Level 6	1	0	0	2.4% (1)
	Level 7	0	1	0	2.4% (1)

**Table 7:** Causality assessment of ADRs in the reference cohort.

Scale	Causality Assessment	Other Antibiotics							Total
		Ceftazidime	Cefuroxime	Piperacillin–Tazobactam	Amoxicillin–Clavulanic Acid	Metronidazole	Cefuroxime–Sulbactam	Ceftriaxone	
**WHO–UMC Criteria**	Certain	0	0	0	0	0	0	0	0
	Probable/Likely	0	1	0	4	0	0	1	46.2% (6)
	Possible	1	0	1	2	2	1	0	53.9% (7)
**Naranjo Probability**	Definite	0	0	0	1	0	0	0	7.7% (1)
	Probable	0	1	0	4	0	0	1	46.2% (6)
	Possible	1	0	1	1	2	1	0	46.2% (6)
**Hartwig’s Severity Assessment**	Level 1	0	0	0	0	0	0	0	0
	Level 2	0	1	1	0	0	1	0	23.1% (3)
	Level 3	1	0	0	5	2	0	1	69.2% (9)
	Level 4a	0	0	0	1	0	0	0	7.7% (1)
	Level 4b	0	0	0	0	0	0	0	0
	Level 5	0	0	0	0	0	0	0	0
	Level 6	0	0	0	0	0	0	0	0
	Level 7	0	0	0	0	0	0	0	0

## References

[1] FDA (2016). Drug Safety Communication: FDA Updates Warnings for Oral and Injectable Fluoroquinolone Antibiotics Due to Disabling Side Effects. http://www.fda.gov/Drugs/DrugSafety/ucm511530.htm.

[2] Stahlmann R., Lode H.M. (2013). Risks associated with the therapeutic use of fluoroquinolones. Expert Opin. Drug Saf..

[3] Brunton L., Chabner B., Knollman B. (2013). Goodman and Gilman’s The Pharmacological Basis of Therapeutics.

[4] Oreagba I.A., Oshikoya K.A., Ogar C., Adefurin A.O., Ibrahim A., Awodele O., Oni Y. (2017). Adverse reactions to fluoroquinolones in the Nigerian population: An audit of reports submitted to the National Pharmacovigilance Centre from 2004 to 2016. Pharmacol. Res. Perspect..

[5] Rubinstein E., Camm J. (2002). Cardiotoxicity of fluoroquinolones. J. Antimicrob. Chemother..

[6] Domagala J.M. (1994). Structure-activity and structure-side-effect relationships for the quinolone antibacterials. J. Antimicrob. Chemother..

[7] Kandasamy A., Srinath D. (2012). Levofloxacin-induced acute anxiety and insomnia. J. Neurosci. Rural Pract..

[8] Liu H.H. (2010). Safety Profile of the Fluoroquinolones. Drug Saf..

[9] Christ W. (1990). Central nervous system toxicity of quinolones: Human and animal findings. J. Antimicrob. Chemother..

[10] (2008). Levofloxacin (Levaquin) Package Insert. https://druginserts.com/lib/rx/meds/levaquin-4/page/10/.

[11] Francis J.K., Higgins E. (2014). Permanent Peripheral Neuropathy: A Case Report on a Rare but Serious Debilitating Side-Effect of Fluoroquinolone Administration. J. Investig. Med. High Impact Case Rep..

[12] FDA (2013). Drug Safety Communication: FDA Requires Label Changes to Warn of Risk for Possibly Permanent Nerve Damage from Antibacterial Fluoroquinolone Drugs Taken by Mouth or by Injection. https://www.fda.gov/downloads/Drugs/DrugSafety/UCM365078.pdf.

[13] Morales D., Pacurariu A., Slattery J., Pinheiro L., McGettigan P., Kurz X. (2019). Association Between Peripheral Neuropathy and Exposure to Oral Fluoroquinolone or Amoxicillin-Clavulanate Therapy. JAMA Neurol..

[14] Cohen J.S. (2001). Peripheral neuropathy associated with fluoroquinolones. Ann. Pharmacother..

[15] Golomb B.A., Koslik H.J., Redd A.J. (2015). Fluoroquinolone-induced serious, persistent, multisymptom adverse effects. BMJ Case Rep..

[16] Mancano M.A. (2014). Ciprofloxacin-Induced Syndrome of Inappropriate Antidiuretic Hormone; Anaphylactic Shock Due to Thrombolytic Administration; Hydroxychloroquine-Induced QT-Interval Prolongation; Complex Regional Pain Syndrome After Tetanus Toxoid Injection. Hosp. Pharm..

[17] Mocan M., Blaga S.N. (2016). Severe Hyponatremia due to Levofloxacin Treatment for *Pseudomonas aeruginosa* Community-Acquired Pneumonia in a Patient with Oropharyngeal Cancer. Case Rep. Med..

[18] Yam F.K., Eraly S.A. (2012). Syndrome of inappropriate antidiuretic hormone associated with moxifloxacin. Am. J. Health Syst. Pharm..

[19] Jones S.C., Sorbello A., Boucher R.M. (2011). Fluoroquinolone-Associated Myasthenia Gravis Exacerbation. Drug Saf..

[20] Forooghian F., Brophy J.M., Bird S.T., Maberley D. (2012). Oral Fluoroquinolones and the Risk of Retinal Detachment. JAMA.

[21] Laxminarayan R., Chaudhury R.R. (2016). Antibiotic Resistance in India: Drivers and Opportunities for Action. PLoS Med..

[22] Yamaguchi H., Kawai H., Matsumoto T., Yokoyama H., Nakayasu T., Komiya M., Shimada J. (2007). Post-Marketing Surveillance of the Safety of Levofloxacin in Japan. Chemotherapy.

[23] Das M., Angeli F., Krumeich A.J.S.M., van Schayck O.C.P. (2018). The gendered experience with respect to health-seeking behavior in an urban slum of Kolkata, India. Int. J. Equity Health.

[24] Lapi F., Tuccori M., Motola D., Pugi A., Vietri M., Montanaro N., Vaccheri A., Leoni O., Cocci A., Leone R. (2010). Safety Profile of the Fluoroquinolones. Drug Saf..

[25] Jose J., Rao P.G.M., Jimmy B. (2008). Adverse drug reactions to fluoroquinolone antibiotics—Analysis of reports received in a tertiary care hospital. Int. J. Risk Saf. Med..

[26] Piscitelli S.C., Spooner K., Baird B., Chow A.T., Fowler C.L., Williams R.R., Natarajan J., Masur H., Walker R.E. (1999). Pharmacokinetics and safety of high-dose and extended-interval regimens of levofloxacin in human immunodeficiency virus-infected patients. Antimicrob. Agents Chemother..

[27] FDA (2015). Joint Meeting of the Antimicrobial Drugs Advisory Committee and the Drug Safety and Risk Management Advisory Committee. https://www.pharmamedtechbi.com/~/media/Supporting%20Documents/The%20Pink%20Sheet%20DAILY/2015/November/Antimicrobial%20AC%20FDA%20briefing%2011515.pdf.

[28] Leone R., Venegoni M., Motola D., Moretti U., Piazzetta V., Cocci A., Resi D., Mozzo F., Velo G., Burzilleri L. (2003). Adverse drug reactions related to the use of fluoroquinolone antimicrobials: An analysis of spontaneous reports and fluoroquinolone consumption data from three Italian regions. Drug Saf..

[29] Kulthanan K., Chularojanamontri L., Manapajon A., Dhana N., Jongjarearnprasert K. (2011). Cutaneous adverse reactions to fluoroquinolones. Dermatitis.

[30] Durey A., Baek Y.S., Park J.S., Lee K., Ryu J.S., Lee J.S., Cheong M.H. (2010). Levofloxacin-Induced Achilles Tendinitis in a Young Adult in the Absence of Predisposing Conditions. Yonsei Med. J..

[31] Connelly S., Bayliff C., Mehta S. (2002). Levofloxacin-induced bilateral Achilles tendinopathy. Can. J. Hosp. Pharm..

[32] Gold L., Igra H. (2003). Levofloxacin-induced tendon rupture: A case report and review of the literature. J. Am. Board Fam. Pract..

[33] Etminan M., Brophy J.M., Samii A. (2014). Oral fluoroquinolone use and risk of peripheral neuropathy: A pharmacoepidemiologic study. Neurology.

[34] Liu X., Ma J., Huang L., Zhu W., Yuan P., Wan R., Hong K. (2017). Fluoroquinolones increase the risk of serious arrhythmias: A systematic review and meta-analysis. Medicine.

[35] Morganroth J., DiMarco J.P., Anzueto A., Niederman M.S., Choudhri S. (2005). A Randomized Trial Comparing the Cardiac Rhythm Safety of Moxifloxacin vs. Levofloxacin in Elderly Patients Hospitalized with Community-Acquired Pneumonia. Chest.

[36] Lapi F., Wilchesky M., Kezouh A., Benisty J.I., Ernst P., Suissa S. (2012). Fluoroquinolones and the Risk of Serious Arrhythmia: A Population-Based Study. Clin. Infect. Dis..

[37] Chodosh S., Lakshminarayan S., Swarz H., Breisch S. (1998). Efficacy and safety of a 10-day course of 400 or 600 milligrams of grepafloxacin once daily for treatment of acute bacterial exacerbations of chronic bronchitis: Comparison with a 10-day course of 500 milligrams of ciprofloxacin twice daily. Antimicrob. Agents Chemother..

[38] Chou H.W., Wang J.L., Chang C.H., Lee J.J., Shau W.Y., Lai M.S. (2013). Risk of Severe Dysglycemia Among Diabetic Patients Receiving Levofloxacin, Ciprofloxacin, or Moxifloxacin in Taiwan. Clin. Infect. Dis..

[39] (2008). FLOXIN® Tablets (Ofloxacin Tablets) Package Insert. https://www.accessdata.fda.gov/drugsatfda_docs/label/2008/019735s059lbl.pdf.

[40] Khaliq Y., Zhanel G.G. (2003). Fluoroquinolone-Associated Tendinopathy: A Critical Review of the Literature. Clin. Infect. Dis..

[41] Shamna M., Dilip C., Ajmal M., Linu Mohan P., Shinu C., Jafer C.P., Mohammed Y. (2014). A prospective study on Adverse Drug Reactions of antibiotics in a tertiary care hospital. Saudi Pharm. J..

[42] Jayanthi C., Reddy N.S. (2017). A profile of adverse drug reactions to antimicrobial agents at a tertiary care hospital. Indian J. Pharm. Pharmacol..

[43] Dhar K., Sinha A., Gaur P., Goel R., Chopra V.S., Bajaj U. (2015). Pattern of adverse drug reactions to antibiotics commonly prescribed in department of medicine and pediatrics in a tertiary care teaching hospital, Ghaziabad. J. Appl. Pharm. Sci..

